# Surgeon-General J. K. Barnes

**Published:** 1879-11-20

**Authors:** 


### *SURGEON-GENERAL J. K. BARNES.*

This eminent and indefatigable man of the United States Army has donated to the Library of the Southern Medical College a number of valuable volumes and circulars, a list of which we append—among them the Medical and Surgical History of the War of the Rebellion. These huge and comprehensive volumes, so replete with valuable statistical information, so wonderfully illustrated, and so elaborately gotten up in detail, evince a most prodigious amount of energy, skill and talent on the part of the government officials and Surgeon-General Barnes, who supervised the work. They will be thankfully appreciated. The following is the list of the books:

Catalogue of Army Medical Museum Surgical Section.

Report on Excisions of the Head of the Femur.

Report on Surgical cases in the Army Circular No. 3.

Report on Epidemic Cholera and Yellow Fever.

Report on Hygiene of the United States Army—Plans and Specifications for Hospitals—Transport of Sick and Wounded.

Four large volumes War of the Rebellion.

An Atlas of Human Anatomy, and a number of valuable circulars.

